# The Next Step

**Published:** 1919-03-22

**Authors:** 


### THE NEXT STEP.

It may seem premature to speak of the consequences of the establishment of a Ministry of Health while the Bill for this purpose is still on the knees of the Parliamentary gods. But as we have previously pointed out, the present Bill is little more than a sketch-plan, and the scheme in practice will be shaped by administrative action. Great powers will be given to the Minister of Health, but how these are to be interpreted and applied will be expressed by Orders in Council. There may be objections to such a fashion of legislation, as the administration of the National Insurance Act abundantly shows. But in existing circumstances it cannot be successfully contested. Parliament is busied about many things, and the country—at least so it is believed—wants reforms in a dozen different directions. Hence the practical legislator has to get "powers," and to leave the exercise of these to a Government department controlled by, or perhaps controlling, a responsible Minister. This at all events is the method adopted in the Bill presented to the House of Commons by Dr. Addison, and as no serious objection has been raised to the scheme, it may be presumed that in the course of a few weeks the Bill will be converted into an Act. Then will come the time for a careful scrutiny of the Minister's detailed proposals, and it must be remembered that as these will appear in the shape of Orders in Council their operation will follow shortly on their definition. No doubt, Parliament has the right to challenge any Order which may be issued, but with equal certainty we may say that it is not in the least likely to take such a step. What the Minister decides, in short, will be effectively and without delay the law of the land.

In view of such a position it is imperative that the medical profession should be cultivating clear ideas on the fashion in which legislation and administration may be utilised to promote the general health of the community and to protect and aid the individual sufferer from accident or disease. Doctors have an interest in this question first as good citizens, and secondly because they must be the instruments by which official regulations will be carried into action. It would be idle to pretend that they may safely leave their interests in the hands of the politicians; much experience has taught them the contrary. But unless they have an agreed scheme of service and are willing to support this by all legitimate means, they will find themselves helpless in the hands of the bureaucracy. Representatives of the profession are to sit on the Advisory Council which is to assist the Minister. Who is to select these representatives? The question is a capital one and it ought to be pressed in Parliament. Unless representation is really what it professes to be it is worse than useless, for it provides a shelter for the Minister against all professional criticism. There are obviously, therefore, abundant reasons why without delay the profession, in contemplating the Government Bill, should apply their minds to a contemplation of the next step.

